# Predicting Postoperative Lung Function in Patients with Lung Cancer Using Imaging Biomarkers

**DOI:** 10.3390/diseases12040065

**Published:** 2024-03-24

**Authors:** Oh-Beom Kwon, Hae-Ung Lee, Ha-Eun Park, Joon-Young Choi, Jin-Woo Kim, Sang-Haak Lee, Chang-Dong Yeo

**Affiliations:** 1 Division of Pulmonary, Critical Care and Sleep Medicine, Department of Internal Medicine, College of Medicine, The Catholic University of Korea, Seoul 06591, Republic of Korea; obkwon@catholic.ac.kr (O.-B.K.); tawoe@naver.com (J.-Y.C.); medkjw70@gmail.com (J.-W.K.); agmante@gmail.com (S.-H.L.); 2 Department of Internal Medicine, Kangwon National University Hospital, Chuncheon 24289, Republic of Korea; 3 Coreline Soft Co., Ltd., Seoul 03991, Republic of Korea; haeung.lee@corelinesoft.com (H.-U.L.); haeun.park@corelinesoft.com (H.-E.P.)

**Keywords:** imaging biomarkers, deep learning, postoperative lung function, lung cancer

## Abstract

There have been previous studies conducted to predict postoperative lung function with pulmonary function tests (PFTs). Computing tomography (CT) can quantitatively measure small airway walls’ thickness, lung volume, pulmonary vessel volume, and emphysema area, which reflect the severity of respiratory diseases. These measurements are considered imaging biomarkers. This study aimed to predict postoperative lung function with imaging biomarkers. A retrospective analysis of 79 patients with lung cancer who had undergone lung surgery was completed. Postoperative lung function measured by forced expiratory volume in one second (FEV_1_) was defined as an outcome. Preoperative clinico-pathological parameters and imaging biomarkers representing airway walls’ thickness, severity of emphysema, total lung volume, and pulmonary vessel volume were measured quantitatively in chest CT by an automated segmentation software, AVIEW COPD. Pi1 was defined as the first percentile along the histogram of lung attenuation that represents the degree of emphysema. Wafw was defined as the airway thickness, which was calculated by the full-width at half-maximum method. Logistic and linear regressions were used to assess these variables. If the actual postoperative FEV_1_ was higher than the postoperative FEV_1_ projected by a formula, the group was considered to be preserved. Among the 79 patients, 16 of the patients were grouped as a non-preserved group, and 63 of them were grouped as a preserved group. The patients in the preserved FEV_1_ group had a higher vessel volume than the non-preserved group. Pi1 and Wafw were independent predictors of postoperative lung function. Imaging biomarkers can be considered significant variables in predicting postoperative lung function in patients with lung cancer.

## 1. Introduction

Chronic obstructive pulmonary disease (COPD) is a major independent risk factor for lung cancer and a common comorbid disease [1]. COPD is characterized by persistent airflow limitation, and a pulmonary function test (PFT) is required to make the diagnosis. The severity of airflow limitation in patients with COPD is evaluated by post-bronchodilator forced expiratory volume in one second (FEV_1_) [2]. However, patients with the same stage of severity based on FEV_1_ have different degrees of emphysema. Due to this limitation and the development of computing tomography (CT) analysis techniques, although PFT has been previously used as a method to evaluate lung function, research using CT as a method to understand the pathophysiology and quantitatively measure the severity of COPD to treat and predict its exacerbations has increased over the past decade [3].

Surgery is the standard therapy for early-stage lung cancer, and postoperative lung function is crucial because it is related to mortality. Therefore, predicting postoperative lung function is important in order to decide treatment modalities. Postoperative FEV_1_ is a variable widely used to represent postoperative lung function. Since patients with lung cancer have COPD as a common comorbid disease, they usually have a limitation of the airways, resulting in a decrease in FEV_1_. Previous studies have estimated predicted postoperative FEV_1_ (ppoFEV_1_) using the following formula: ppoFEV_1_ = preoperative FEV_1_ × (19-segments to be removed)/19 [4,5]. A meta-analysis has shown that CT volume and density measures are the preferred method for predicting postoperative FEV_1_ [6]. In our previous study [7], we showed that preoperative residual volume (RV)/total lung capacity (TLC) is positively correlated with postoperative lung function. As a result, in addition to the number of segments removed, lung function should also be taken into account because the concurrent respiratory condition affects postoperative lung function. Conventionally, PFT has been used as a method to evaluate lung function, but there is growing evidence that imaging modalities such as CT can also estimate lung function [6].

Due to the development of deep learning, the airway tree segmentation method in chest CT has been improved [8]. After airway segmentation, it is feasible to evaluate the emphysema region by calculating the low-attenuation area, measuring the thickness of the airway wall, the total lung volume, and the total vascular volume. These variables are considered imaging biomarkers. The aim of this study was to determine whether imaging biomarkers can be used as variables in predicting postoperative lung function in patients with lung cancer. 

## 2. Materials and Methods

### 2.1. Patients 

We retrospectively reviewed 94 patients with lung cancer who had undergone lung surgery at Eunpyoung St. Mary’s Hospital between July 2016 and February 2021. Four patients were excluded due to missing PFT values. Eleven patients were excluded because their CT scans were not compatible with the software used in this study. Thus, a total of 79 patients were finally included in this study. 

### 2.2. AI Algorithm 

A deep learning algorithm (AVIEW COPD, Coreline Soft, Seoul, South Korea), available commercially, was utilized to examine the imaging biomarkers obtained from the CT scans. The algorithm was implemented to perform airway segmentation using the 2.5D convolutional neural net (2.5D CNN), which was trained and evaluated by CT scans obtained from the Korean obstructive lung disease study [8]. This automated segmentation software significantly improved branch level segmentation accuracy [9]. 

### 2.3. Chest CT Acquisition 

The CT scanners used in this study were CT scanners manufactured by Seimens Healthineers (Somatom Definition Edge; Forchheim, Germany), GE Healthcare (Revolution ACT; Chicago, IL, USA), and Philips (Brilliance 64 CT; Amsterdam, The Netherlands). The parameters of the Somatom Definition Edge scanner were 90–130 kVP, 65–308 mAs, and a slice thickness of 3 mm. The parameters of the Revolution ACT scanner were 100 and 120 kVP, 1–12 mAs, and a slice thickness of 1.25 and 2.5 mm. The parameters of the Brillance 64 CT machine were 120 kVP, 166–172 mAs, and a slice thickness of 5 mm. 

### 2.4. Variables’ Explanation and Outcome Definition 

The patients’ age, sex, pathology type, tumor stage according to the eighth tumor node metastasis (TNM) classification and location, operation type, treatment modalities, and preoperative PFT values, including FEV_1_, FEV_1_/FVC, RV/TLC, and diffusing capacity of the lung for carbon monoxide (DL_CO_), were collected. The patients were classified as non-smokers if they had never smoked or had smoked less than 100 cigarettes in their lives and as ever smokers if they had smoked more than 100 cigarettes. All the PFT values were measured before the inhalation of a bronchodilator. The operation type was divided into two groups—video-assisted thoracic surgery (VATS) and open surgery. Neoadjuvant chemotherapy, adjuvant chemotherapy, palliative chemotherapy, neoadjuvant radiotherapy, adjuvant radiotherapy, and palliative radiation were all used as treatment methods. In this study, we considered imaging biomarkers, which are quantitatively measured in chest CT, as the variables. Emphysema, airway thickness, total lung volume, and pulmonary vessel volume were measured quantitatively in the chest CT using an automatic segmentation software (AVIEW COPD, Corelinesoft, Seoul, Republic of Korea). 

Pulmonary emphysema is defined as an abnormal permanent enlargement of airspaces distal to the terminal bronchioles [10,11] and results in low-attenuated areas (LAA) in a chest CT that are below −950 Hounsefield unit (HU) [3]. Since the extent of emphysema in CT is associated with pulmonary function decline [12], the variables representing emphysema were evaluated. The percentage of LAA compared to the whole lung measured in cubic centimeters (cc) was defined as the variable LAAsize for the quantification of emphysema. Two variables, Pi1 and Pi15, which correspond to the first and fifteenth percentiles along the histogram of lung attenuation, were defined as an alternative method to quantify emphysema [13]. 

The remodeling of small airways, which have an internal diameter smaller than 2 mm, is known to represent airflow limitation in patients with COPD. Through the remodeling process of these airways, the airways become thicker, and the thickness of the airways is strongly associated with the progression of COPD [14]. However, due to the limited resolution of CT scans, small airways are difficult to measure. Nakano et al. overcame this obstacle by showing that relatively larger airways measured by CT reflect small airways measured histologically [15]. In order to represent airway thickness, three variables—Wafw, Waband, and Pi10fw—were measured. The full-width half-maximum (FWHM) method is the most frequently used technique to measure airways quantitatively. The inner and outer margins of the airway wall were measured using linear rays from the airway center. The airway thickness was measured by the difference between the half point between the minimum and maximum gray levels and the maximum point of the gray level [16,17]. The proportion of the mean of the airway wall area calculated by the FWHM method was defined as Wafw. However, the FWHM method overestimates the wall dimensions in small airways [17]. The integral-based half-band (IBHB) method, which is a threshold-based method, was developed and showed better correlation with pulmonary functions [18]. The mean of the airway wall area calculated by the IBHB method was defined as Waband. Another parameter, Pi10, was developed. It was derived by plotting the square root of the airway wall area against the internal perimeter of each measured airway. After creating a regression line, Pi10 was defined as the square root of the airway wall area with an internal perimeter of 10 mm on the regression line [19]. The airway wall area was calculated by the FWHM method, and the corresponding Pi10 value was defined as Pi10fw. 

The total lung volume, measured in cc, was defined as TLV. The total airway count, which quantifies the total number of visible airways from the pulmonary tree, was defined as TAC. It is well known that pulmonary lung function and pulmonary vasculature are correlated. Park et al. showed that, as emphysema severity increased, the number of pulmonary vessels decreased [20]. The total vessel volume, which quantifies the total vessel volume, including pulmonary arteries and pulmonary veins, was defined as VV. 

The pre-bronchodilator value of FEV_1_ (%) measured at 6 ± 3 months after surgery was defined as the outcome. In order to analyze the characteristics of patients who have a better lung function than predicted after surgery, the patients were categorized into two groups. The patients were grouped into the FEV_1_-preserved group if they had a higher FEV_1_ than the ppoFEV_1_ and into the FEV_1_-non-preserved group otherwise. Linear and logistic regressions were performed to analyze the variables. *p* values < 0.05 were indicated as showing statistical significance. Statistical analyses were performed using R (version 4.0.2; The R foundation, Vienna, Austria). 

## 3. Results

### 3.1. Overall Patient Characteristics 

A total of 79 patients with primary lung cancer who had undergone surgery with mediastinal lymph node dissection were included in this study. The patients’ characteristics are presented in Table 1. The mean age of the patients was 69.18 years, and the majority of the patients were male (64.56%). A total of 36 patients (45.57%) were never smokers, and 43 patients (54.43%) were smokers. Regarding the operation method, 26 patients (32.91%) received open surgery, and 53 patients (67.09%) received VATS. Stage I was the most common stage (78.48%), and the right upper lung was the most common site of the cancer (34.18%). About 22.78% of the patients had received adjuvant chemotherapy. The preoperative PFT results were measured before bronchodilator usage, and the imaging biomarkers were measured using the automatic software AVIEW COPD. 

### 3.2. Comparison between FEV_1_-Non-Preserved Groups and -Preserved Groups 

The comparison between the FEV_1_-non-preserved and -preserved groups are provided in Table 2. The age, sex, histologic features, smoking history, operation type, stage, location, and preoperative PFT values did not differ between the two groups. The mean value of the total vessel volume was significantly higher in the FEV_1_-preserved group than in the non-preserved group (113.76 ± 46.7 and 89.99 ± 33.45, respectively with *p* value 0.027). 

### 3.3. Factors Associated with Preserved Postoperative FEV_1_

Table 3 displays the findings of the logistic regression analysis that determined the variables linked to postoperative FEV_1_ preservation. The outcome was defined as positive if the postoperative FEV_1_ was higher than the ppoFEV_1_, which means that it is “preserved” and is negative otherwise. Adjuvant chemotherapy (unadjusted odds ratio (OR) 0.392, 95% confidence interval (CI) 0.106–9.779, *p* = 0.124), Pi10fw (unadjusted OR 0.379, 95% CI 0.086–1.673, *p* = 0.2), TAC (unadjusted OR 1.006, 95% CI 0.997–1.016, *p* = 0.176), and VV (unadjusted OR 1.015, 95% CI 0.999–1.031, *p* = 0.063) had *p* values less than 0.2 in the univariate analysis that were entered into the multivariate analysis. None of the variables were statistically significant in the multivariate logistic regression analysis. 

The results of the linear regression are provided in Table 4. Since age is a strong factor which is associated with postoperative lung function, it was entered into multiple linear regressions with variables that had *p* values less than 0.2. The multiple linear regression model had an R^2^ value of 0.134 and a *p* value of 0.024. Pi1 and Wafw remained statistically significant. Pi1 was positively correlated, and Wafw was negatively associated.

### 3.4. Comparison of Conventional Formula and Multiple Linear Regression Model 

**Figure 1:**
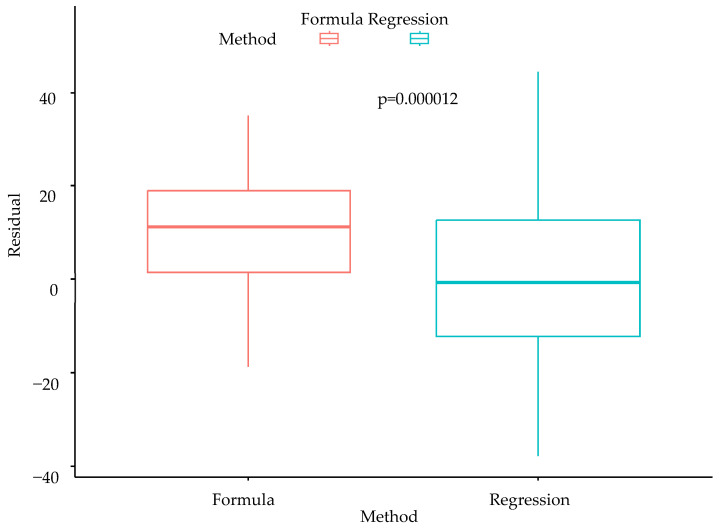
Comparison of conventional formula and multiple linear regression model in predicting postoperative lung function, shown with residual box plots.

Residual box plots of the conventional formula model and the multiple linear regression model are presented in Figure 1. The residual was defined as the difference between the predicted and the actual postoperative FEV_1_. In the conventional formula model, the ppoFEV_1_ was calculated using the conventional formula. In the multiple linear regression model, the ppoFEV_1_ was calculated using the following formula: ppoFEV_1_ = 373.8 + 0.28 × age + 0.156 × Pi1 − 2.111 × Wafw − 0.001 × TLV. The value of intercept in this model was 373.8. 

## 4. Discussion

In this study, we used imaging biomarkers to predict postoperative lung function. The total vessel volume, including pulmonary arteries and vessels, was higher in the FEV_1_-preserved group. The Pi1, the first percentile of the low-attenuating area, was positively correlated, and the wall area (% ratio of the wall area to the airway area) measured by the FWHM method was negatively correlated with the postoperative FEV_1_. We developed a novel formula for multivariate linear regression utilizing these variables. We compared this formula with the conventional formula, which uses the number of lung segments removed by the residual box plot. The residual was defined as the difference between the actual postoperative FEV_1_ and the formula-predicted postoperative FEV_1_. The residual mean and the interquartile box were closer to zero in our new formula, showing the promising effects of radiological biomarkers in predicting postoperative lung function. 

Approximately 40–70% of patients with lung cancer have coexisting COPD [1]. An important pathophysiological characteristic of COPD is pulmonary vascular alteration. In the patients with severe COPD, there was a decreased ratio of the cross-sectional area of tiny pulmonary arteries to the overall area of the lung [21]. As the severity of emphysema based on chest CT imaging increased, the number of pulmonary vessels decreased [20]. A previous study has shown the pathophysiology of this pulmonary vascular change. In said study, the vascular endothelial growth factor (VEGF), which induces endothelial cell growth, and the vascular endothelial growth factor receptor 2 (VEGFR2) were significantly decreased in emphysematous lungs [18]. Our study was consistent with these previous studies. The patients in the FEV_1_-non-preserved group had lower total pulmonary vessel volumes, measured with the AVIEW COPD system. This indicated that patients with a severe form of emphysema tend to have a lower postoperative lung function than expected. 

Emphysema replaces the normal lung with air-containing spaces, resulting in CT attenuation. A value of -950 HU is usually used as the threshold to identify emphysema lesions. To avoid noise in CT measurements and obtain robust results, the percentile approach was introduced as an alternative. Emphysema quantification was measured by CT attenuation at a given percentile along the histogram of lung attenuation [3,22]. In our study, Pi1, the first percentile of the low-attenuating area, was positively correlated with postoperative lung function. Since the severity of emphysema was lower in the patients with a higher Pi1, the patients with less-severe emphysema had a higher postoperative lung function. 

The progression of COPD is associated with inflammatory mucous exudates in the lumen and infiltration of the wall by inflammatory immune cells resulting in the thickening of the airway’s walls [14]. Han et al. showed that a greater airway wall thickness is associated with COPD exacerbation frequency [23]. Wafw, an imaging biomarker representing airway wall thickness, was negatively correlated with postoperative lung function in our study. This result was consistent with previous studies [3]. Waband and Pi10fw, two further radiological indicators of airway wall thickness, failed to reach statistical significance. 

The total lung volume in patients with COPD is increased due to expiratory airflow limitation [24]. The total lung volume was found to be inversely linked with the outcome in a single linear regression analysis, meaning that individuals with greater lung volumes had a lower postoperative lung function than expected (*p* = 0.159). However, the total lung volume was not statistically significant in multiple linear regressions (*p* = 0.656). 

Overall, the multiple linear regression model with imaging biomarkers showed a better performance than the conventional formula in terms of predicting postoperative lung function. Widely used conventional formulas use preoperative FEV_1_ and the number of segments resected as the variables [25]. PFT results, including FEV_1_, are routinely used to evaluate lung function, but there are some limitations. Elderly patients or patients with a general weakness might not perform PFT correctly [26]. The volume of each lung segment can also differ. Contrary to this variability, imaging biomarkers are quantified in chest CT, which offers repeatable and reliable data, with reduced variations, for those who are unable to perform PFT. 

Regarding imaging biomarkers, airway tree segmentation is important because it quantifies anatomical features. In our study, segmentation was performed via a semi-automated method, using AVIEW COPD. Segmentation was performed voxel-by-voxel by a 2.5D CNN and trained in a supervised manner. The 2.5D patches captured the 3D structure of the airway tree efficiently. Since this method has been validated on multiple datasets, these imaging biomarkers are highly reliable [8]. Therefore, imaging biomarkers can be considered important factors in predicting postoperative lung function. 

However, our study has several limitations. First, the number of patients enrolled was relatively small. Second, excluding patients without postoperative FEV_1_ might have led to selection bias. Patients with postoperative FEV_1_ may present substantially reduced lung function since PFT is not frequently performed, and physicians typically only perform PFT when patients have symptoms. Future studies are required with a large number of patients, and PFT should be performed routinely to avoid selection bias.

## 5. Conclusions

Imaging biomarkers can be considered significant variables in predicting postoperative lung function in patients with lung cancer. The total pulmonary vessel volume was higher in the lung function-preserved group. Pi1, an imaging biomarker representing emphysema, was positively correlated, and Wafw, an imaging biomarker representing airway wall thickness, was negatively correlated with postoperative lung function. 

## Figures and Tables

**Table 1:** Clinical characteristics of the study patients.

	Overall Patients (*n* = 79)
Age (years) mean ± SD	69.18 ± 7.89
Sex, *n* (%)	
Male	51 (64.56)
Female	28 (35.44)
Histologic features, *n* (%)	
Adenocarcinoma	55 (69.62)
Squamous cell carcinoma	20 (25.32)
Others	4 (5.06)
Smoking, *n* (%)	
Never smoker	36 (45.57)
Ever smoker	43 (54.43)
Operation, *n*(%)	
Open	26 (32.91)
VATS	53 (67.09)
Stage, *n* (%)	
I	62 (78.48)
II	8 (10.13)
III	9 (11.39)
IV	0
Location, *n* (%)	
RUL	27 (34.18)
RML	4 (5.06)
RLL	19 (24.05)
LUL	23 (29.11)
LLL	6 (7.59)
Therapy	
Adjuvant chemotherapy, n (%)	18 (22.78)
Palliative chemotherapy, n (%)	5 (6.33)
Adjuvant radiotherapy, n (%)	5 (6.33)
Palliative radiotherapy, n (%)	2 (2.53)
PFT	
FEV_1_ (pre) (%), mean ± SD	101.99 ± 18.62
FEV_1_/FVC (pre) (%), mean ± SD	70.46 ± 8.57
DL_CO_ (pre) (%), mean ± SD	98.23 ± 18.89
RV/TLC (pre) (%), mean ± SD	36.49 ± 8.99
Radiological biomarkers	
Pi1 (HU) mean ± SD	−934.30 ± 33.36
Pi15 (HU) mean ± SD	−890.30 ± 30.50
Pi10fw (mm) mean ± SD	5.00 ± 0.54
Wafw (%) mean ± SD	71.99 ± 2.99
Waband (%) mean ± SD	68.99 ± 14.12
LAAsize (%) mean ± SD	1.40 ± 2.77
TAC (ea) mean ± SD	141.41 ± 88.45
VV (cc) mean ± SD	108.95 ± 45.18
TLV (cc) mean ± SD	4216.28 ± 1098.24

Sqcc, squamous cell carcinoma; RUL, right upper lobe; V0, preoperative; FEV1, forced expiratory volume in one second; FVC, forced vital capacity; DLco, diffusing capacity of the lung for carbon monoxide; RV, residual volume; TLC, total lung capacity; Pi10fw, internal perimeter of the segment of the bronchus measured in the full-width at half-maximum method; LAAsize, low-attenuation area size; Pi1, first percentile Hounsefield unit of the low-attenuation area of the lung; Pi15, fifteenth percentile Hounsefield unit of the low-attenuation area of the lung; TAC, total airway count; VV, total vessel volume; and TLV, total lung volume.

**Table 2:** Comparison between FEV_1_-non-preserved and -preserved groups.

	FEV_1_-Non-Preserved (n = 16, 20.25%)	FEV_1-_Preserved (n = 63, 79.75%)	*p* Value
Age (years) mean ± SD	67.31 ± 5.99	69.65 ± 8.28	0.209
Sex, *n* (%)			0.437
Male	9 (56.2)	42 (66.7)	
Female	7 (43.8)	21 (33.3)	
Histologic features, *n* (%)			0.187
Sqcc.	2 (12.5)	18 (28.6)	
Non-Sqcc	14 (87.5)	45 (71.4)	
Smoking, *n* (%)			0.690
Never smoker	8 (50.0)	28 (44.4)	
Ever smoker	8 (50.0)	35 (55.6)	
Operation, *n* (%)			0.451
Open	4 (25.0)	22 (34.9)	
VATS	12 (75.0)	41(65.1)	
Stage, *n* (%)			0.704
I	12 (75.0)	50 (79.4)	
II–III	4 (25.0)	13 (20.6)	
Location, *n* (%)			0.277
BUL	12 (75.0)	38 (60.3)	
Other lobes	4 (25.0)	25 (39.7)	
PFT			
FEV1 (pre) (%), mean ± SD	100.94 ± 13.71	102.25 ± 19.76	0.758
FEV1/FVC (pre (%), mean ± SD	72.31 ± 7.02	69.98 ± 8.90	0.273
DL_CO_ (pre) (%), mean ± SD	97.50 ± 11.80	98.42 ± 20.39	0.816
RV/TLC (pre) (%), mean ± SD	36.38 ± 9.19	36.52 ± 9.01	0954
Radiological biomarkers			
Pi1 (HU) mean ± SD	−934.56 ± 44.20	−934.24 ± 30.46	0.978
Pi15 (HU) mean ± SD	−886.44 ± 40.68	−891.29 ± 27.66	0.657
Pi10fw (cc) mean ± SD	5.24 ± 1.07	4.94 ± 0.26	0.288
Wafw (%) mean ± SD	71.36 ± 3.04	72.15 ± 2.97	0.366
Waband (%) mean ± SD	60.75 ± 24.82	71.08 ± 8.94	0.121
LAAsize (%) mean ± SD	1.55 ± 2.64	1.36 ± 2.83	0.807
TAC (ea) mean ± SD	115.31 ± 61.56	148.03 ± 93.30	0.100
VV (cc) mean ± SD	89.99 ± 33.45	113.76 ± 46.70	0.027
TLV (cc) mean ± SD	4242.42 ± 1224.82	4209.64 ± 1074.34	0.922

Sqcc, squamous cell carcinoma; RUL, right upper lobe; V0, preoperative; FEV1, forced expiratory volume in one second; FVC, forced vital capacity; DLco, diffusing capacity of the lung for carbon monoxide; RV, residual volume; TLC, total lung capacity; Pi10fw, internal perimeter of the segment of the bronchus measured in the full-width at half-maximum method; LAAsize, low-attenuation area size; Pi1, first percentile Hounsefield unit of the low-attenuation area of the lung; Pi15, fifteenth percentile Hounsefield unit of the low-attenuation area of the lung; TAC, total airway count; VV, total vessel volume; and TLV, total lung volume.

**Table 3:** Factors associated with preserved postoperative FEV1 using logistic regression.

	Univariable Analysis			Multivariable Analysis		
	OR	95% CI	*p* Value	OR	95% CI	*p* Value
Sex (Male vs. Female)	1.556	0.509–4.758	0.439			
Age	1.038	0.968–1.113	0.291			
Histology (Sqcc. vs. Non sqcc.)	2.800	0.577–13.582	0.201			
Smoking (Ever vs. Never)	1.250	0.417–3.750	0.691			
Stage (II–III vs. I)	0.780	0.216–2.820	0.705			
Location (BUL vs. Other)	0.507	0.147–1.745	0.282			
Operation (Open vs. VATS)	1.610	0.464–5.588	0.453			
V0FEV_1_	1.004	0.975–1.034	0.799			
V0FVC	1.009	0.970–1.048	0.663			
V0FEV_1_/FVC	0.965	0.898–1.037	0.331			
V0DLco	1.002	0.973–1.033	0.861			
RV/TLC (≥40% vs. <40%)	0.496	0.153–1.608	0.243			
Adjuvant chemotherapy	0.392	0.119–1.291	0.124	0.412	0.118–1.440	0.164
Palliative chemotherapy	0.350	0.053–2.297	0.274			
Adjuvant radiotherapy	1.017	0.106–9.779	0.988			
Palliative radiotherapy	0.242	0.014–4.094	0.325			
Pi1	1.000	0.984–1.017	0.972			
Pi15	0.995	0.978–1.012	0.569			
Pi10fw	0.379	0.086–1.673	0.200	0.445	0.113–1.747	0.246
Wafw	1.087	0.912–1.294	0.351			
LAAsize	0.977	0.808–1.181	0.811			
TAC	1.006	0.997–1.016	0.167	0.999	0.989–1.009	0.865
VV	1.015	0.999–1.031	0.063	1.015	0.995–1.035	0.144
TLV	1.000	0.999–1.001	0.915			

OR; odds ratio; CI; confidence interval; Sqcc, squamous cell carcinoma; BUL, both upper lobes; V0, preoperative; FEV1, forced expiratory volume in one second; FVC, forced vital capacity; DLco, diffusing capacity of the lung for carbon monoxide; RV, residual volume; TLC, total lung capacity; Pi10fw, internal perimeter of the segment of the bronchus measured in the full-width at half-maximum method; LAAsize, low-attenuation area size; Pi1, first percentile Hounsefield unit of the low-attenuation area of the lung; Pi15, fifteenth percentile Hounsefield unit of the low-attenuation area of the lung; TAC, total airway count; VV, total vessel volume; and TLV, total lung volume.

**Table 4:** Factors associated with postoperative FEV_1_ using linear regression.

	Univariate Analysis		Multivariate Analysis (Adjusted R^2^ = 0.134), *p* Value 0.024		
	β ± SE	*p* Value	β ± SE	VIF	*p* Value
Age	0.253 ± 0.263	0.339	0.280 ± 0.259	1.070	0.283
Pi1	0.094 ± 0.671	0.132	0.156 ± 0.075	1.619	0.042
Pi15	0.084 ± 0.068	0.215			
Pi10fw	−2.06 ± 3.88	0.596	−		
Wafw	−1.219 ± 0.686	0.080	−2.111 ± 0.737	1.237	0.005
LAAsize	−0.914 ± 0.746	0.244			
TAC	0.018 ± 0.024	0.446			
VV	−0.027 ± 0.046	0.563			
TLV	−0.003 ± 0.002	0.159	−0.001 ± 0.002	1.440	0.656

β, estimate; SE, standard error; VIF, variance inflation factor; V0, preoperative; FEV1, forced expiratory volume in one second; FVC, forced vital capacity; DLco, diffusing capacity of the lung for carbon monoxide; RV, residual volume; TLC, total lung capacity; Pi10fw, internal perimeter of the segment of the bronchus measured in the full-width at half-maximum method; LAAsize, low-attenuation area size; Pi1, first percentile of the Hounsefield unit of the lung; and Pi15, fifteenth percentile of the Hounsefield unit of the lung.

## Data Availability

The data presented in this study are available upon request from the corresponding author.

## References

[1] Young R.P., Hopkins R.J., Christmas T., Black P.N., Metcalf P., Gamble G.D. (2009). COPD prevalence is increased in lung cancer, independent of age, sex and smoking history. Eur. Respir. J..

[2] Vestbo J., Hurd S.S., Agustí A.G., Jones P.W., Vogelmeier C., Anzueto A., Barnes P.J., Fabbri L.M., Martinez F.J., Nishimura M. (2013). Global strategy for the diagnosis, management, and prevention of chronic obstructive pulmonary disease: GOLD executive summary. Am. J. Respir. Crit. Care Med..

[3] Lynch D.A. (2014). Progress in Imaging COPD, 2004–2014. Chronic Obs. Pulm. Dis..

[4] Cukic V. (2012). Preoperative prediction of lung function in pneumonectomy by spirometry and lung perfusion scintigraphy. Acta Inform. Med..

[5] British Thoracic Society Society of Cardiothoracic Surgeons of Great Britain Ireland Working Party (2001). BTS guidelines: Guidelines on the selection of patients with lung cancer for surgery. Thorax.

[6] Oswald N.K., Halle-Smith J., Mehdi R., Nightingale P., Naidu B., Turner A.M. (2019). Predicting Postoperative Lung Function Following Lung Cancer Resection: A Systematic Review and Meta-analysis. EClinicalMedicine.

[7] Kwon O.B., Yeo C.D., Lee H.Y., Kang H.S., Kim S.K., Kim J.S., Park C.K., Lee S.H., Kim S.J., Kim J.W. (2021). The Value of Residual Volume/Total Lung Capacity as an Indicator for Predicting Postoperative Lung Function in Non-Small Lung Cancer. J. Clin. Med..

[8] Yun J., Park J., Yu D., Yi J., Lee M., Park H.J., Lee J.G., Seo J.B., Kim N. (2019). Improvement of fully automated airway segmentation on volumetric computed tomographic images using a 2.5 dimensional convolutional neural net. Med. Image Anal..

[9] Choe J., Lee S.M., Hwang H.J., Lee S.M., Yun J., Kim N., Seo J.B. (2022). Artificial Intelligence in Lung Imaging. Semin. Respir. Crit. Care Med..

[10] The Definition of Emphysema (1985). Report of a National Heart, Lung, and Blood Institute, Division of Lung Diseases workshop. Am. Rev. Respir. Dis..

[11] Choi H., Kim H., Jin K.N., Jeong Y.J., Chae K.J., Lee K.H., Yong H.S., Gil B., Lee H.J., Lee K.Y. (2022). A Challenge for Emphysema Quantification Using a Deep Learning Algorithm With Low-dose Chest Computed Tomography. J. Thorac. Imaging.

[12] Mohamed Hoesein F.A., de Hoop B., Zanen P., Gietema H., Kruitwagen C.L., van Ginneken B., Isgum I., Mol C., van Klaveren R.J., Dijkstra A.E. (2011). CT-quantified emphysema in male heavy smokers: Association with lung function decline. Thorax.

[13] Heussel C.P., Herth F.J., Kappes J., Hantusch R., Hartlieb S., Weinheimer O., Kauczor H.U., Eberhardt R. (2009). Fully automatic quantitative assessment of emphysema in computed tomography: Comparison with pulmonary function testing and normal values. Eur. Radiol..

[14] Hogg J.C., Chu F., Utokaparch S., Woods R., Elliott W.M., Buzatu L., Cherniack R.M., Rogers R.M., Sciurba F.C., Coxson H.O. (2004). The nature of small-airway obstruction in chronic obstructive pulmonary disease. N. Engl. J. Med..

[15] Nakano Y., Wong J.C., de Jong P.A., Buzatu L., Nagao T., Coxson H.O., Elliott W.M., Hogg J.C., Paré P.D. (2005). The prediction of small airway dimensions using computed tomography. Am. J. Respir. Crit. Care Med..

[16] Kim N., Seo J.B., Song K.S., Chae E.J., Kang S.H. (2008). Semi-automatic measurement of the airway dimension by computed tomography using the full-width-half-maximum method: A study on the measurement accuracy according to the CT parameters and size of the airway. Korean J. Radiol..

[17] Cho Y.H., Seo J.B., Lee S.M., Lee S.M., Choe J., Lee D., Kim N. (2018). Quantitative CT Imaging in Chronic Obstructive Pulmonary Disease: Review of Current Status and Future Challenges. J. Korean Soc. Radiol..

[18] Kasahara Y., Tuder R.M., Cool C.D., Lynch D.A., Flores S.C., Voelkel N.F. (2001). Endothelial cell death and decreased expression of vascular endothelial growth factor and vascular endothelial growth factor receptor 2 in emphysema. Am. J. Respir. Crit. Care Med..

[19] Grydeland T.B., Dirksen A., Coxson H.O., Pillai S.G., Sharma S., Eide G.E., Gulsvik A., Bakke P.S. (2009). Quantitative computed tomography: Emphysema and airway wall thickness by sex, age and smoking. Eur. Respir. J..

[20] Park S.W., Lim M.-N., Kim W.J., Bak S.H. (2022). Quantitative assessment the longitudinal changes of pulmonary vascular counts in chronic obstructive pulmonary disease. Respir. Res..

[21] Yang T., Chen C., Chen Z. (2021). The CT pulmonary vascular parameters and disease severity in COPD patients on acute exacerbation: A correlation analysis. BMC Pulm. Med..

[22] Dirksen A. (2008). Monitoring the progress of emphysema by repeat computed tomography scans with focus on noise reduction. Proc. Am. Thorac. Soc..

[23] Han M.K., Kazerooni E.A., Lynch D.A., Liu L.X., Murray S., Curtis J.L., Criner G.J., Kim V., Bowler R.P., Hanania N.A. (2011). Chronic obstructive pulmonary disease exacerbations in the COPDGene study: Associated radiologic phenotypes. Radiology.

[24] Biselli P., Grossman P.R., Kirkness J.P., Patil S.P., Smith P.L., Schwartz A.R., Schneider H. (2015). The effect of increased lung volume in chronic obstructive pulmonary disease on upper airway obstruction during sleep. J. Appl. Physiol..

[25] Zeiher B.G., Gross T.J., Kern J.A., Lanza L.A., Peterson M.W. (1995). Predicting postoperative pulmonary function in patients undergoing lung resection. Chest.

[26] Koo H.J., Lee S.M., Seo J.B., Lee S.M., Kim N., Oh S.Y., Lee J.S., Oh Y.M. (2019). Prediction of Pulmonary Function in Patients with Chronic Obstructive Pulmonary Disease: Correlation with Quantitative CT Parameters. Korean J. Radiol..

